# The Feasibility of Encapsulated Embryonic Medullary Reticular Cells to Grow and Differentiate Into Neurons in Functionalized Gelatin-Based Hydrogels

**DOI:** 10.3389/fmats.2018.00040

**Published:** 2018-06-28

**Authors:** Ana M. Magariños, Sara Pedron, Marc Creixell, Murat Kilinc, Inna Tabansky, Donald W. Pfaff, Brendan A. C. Harley

**Affiliations:** 1 Laboratory of Neurobiology and Behavior, The Rockefeller University, New York, NY, United States,; 2 Carl R. Woese Institute for Genomic Biology, University of Illinois at Urbana-Champaign, Urbana, IL, United States,; 3 Department of Chemical and Biomolecular Engineering, University of Illinois at Urbana-Champaign, Urbana, IL, United States

**Keywords:** 3D cell culture, hyaluronic acid, neurons, nucleus gigantocellularis, brain development, biomaterial models, gelatin hydrogels

## Abstract

The study of the behavior of embryonic neurons in controlled *in vitro* conditions require methodologies that take advantage of advanced tissue engineering approaches to replicate elements of the developing brain extracellular matrix. We report here a series of experiments that explore the potential of photo-polymerized gelatin hydrogels to culture primary embryonic neurons. We employed large medullary reticular neurons whose activity is essential for brain arousal as well as a library of gelatin hydrogels that span a range of mechanical properties, inclusion of brain-mimetic hyaluronic acid, and adhesion peptides. These hydrogel platforms showed inherent capabilities to sustain neuronal viability and were permissive for neuronal differentiation, resulting in the development of neurite outgrowth under specific conditions. The maturation of embryonic medullary reticular cells took place in the absence of growth factors or other exogenous bioactive molecules. Immunocytochemistry labeling of neuron-specific tubulin confirmed the initiation of neural differentiation. Thus, this methodology provides an important validation for future studies of nerve cell growth and maintenance.

## INTRODUCTION

Large reticular formation neurons in the medulla, neurons in the nucleus gigantocellularis (NGC), have recently been identified as master cells responsible for the arousal of the mammalian brain (Calderon et al., 2016). NGC neurons are located just above the spinal cord and are essential for supporting generalized CNS arousal (Pfaff, 2006), responsible for facilitating the initiation and vigor of all motivated behaviors. They may have evolved from the large Mauthner cells similarly placed in the fish hindbrain and similarly responsible for the rapid initiation of behavior (Pfaff et al., 2014). These nerve cells have extremely wide dendritic arbors and are able to respond to stimuli in all sensory modalities tested (Martin et al., 2010, 2011). Their axonal distribution encompasses ascending projections to the thalamus and hypothalamus and descending projections to all levels of the spinal cord, bilaterally (Jones and Yang, 1985). Their increase in excitability activates the electroencephalogram in a deeply anesthetized animal (Wu et al., 2007) and their growing ability to fire trains of action potentials as a mouse pup grows from postnatal day 3 to postnatal day 6 is correlated with the increased behavioral arousal of the mouse pup during those 4 days (Liu et al., 2016). As a result, increasingly detailed studies of these neurons under controlled conditions may yield significant new information to help understand their roles in brain function and response to injury. However, such studies are difficult in traditional *in vivo* settings.

It is generally acknowledged that standard 2D *in vitro* systems lack important architectural features of native tissues. Tissue engineering approaches have increasingly been used to recreate a myriad of tissue microenvironments in the context of tissue regeneration or for the development of *in vitro* model systems for the study of biological processes, personalized medicine, and pharmacological testing (Neves et al., 2016; Pradhan et al., 2016; Caballero et al., 2017). However, fabrication of ex vivo cerebral tissue has been elusive, due to the high complexity, including multiple hierarchical levels of interconnected neuronal networks. Tumor models have been described within hydrogel matrices (Xiao et al., 2017) and neural cells have been encapsulated successfully within synthetic (Mckinnon et al., 2013), natural (Palazzolo et al., 2015; Alessandri et al., 2016), and protein (Lampe et al., 2013) hydrogels. Besides the use of bulk hydrogels, more sophisticated fabrication techniques have been used such as three-dimensional printing (Gu et al., 2016), lithography (Gurkan et al., 2013), and compartmentalized sca olds (Tang-Schomer et al., 2014). Organoids of certain regions of the brain have been described (Di Lullo and Kriegstein, 2017) but they lack the oxygen and nutrient diffusion provided by biocompatible hydrogel matrices. In order to study the development of these NGC neurons, complementary to *in vivo* studies, 3-dimensional growth platforms may be an ideal framework that could mimic features of the brain’s unique extracellular matrix.

Hydrogel technologies have become increasingly well developed, with recent efforts beginning to utilize a series of hydrogel platforms to study features of brain tumor such as primary glioblastoma (GBM) or tumor metastasis to the brain (Rape et al., 2014; Heffernan et al., 2015; Kingsmore et al., 2016; Chung et al., 2017; Pedron et al., 2017a,b). Recent efforts in our lab have focused on the development of a class of photopolymerizable gelatin hydrogels. Gelatin is a natural polymer that provides cell binding sites (e.g., RGD) and degradation moieties (e.g., MMP sensitive). The functionalization with methacrylamide groups allows for the formation of a covalently crosslinked gel (Pedron and Harley, 2013). Hyaluronic acid (HA) is the major component in the fetal mammalian brain ECM (Baier et al., 2007) and it has been used to culture neuronal cells (Seidlits et al., 2010). Efforts in our lab have demonstrated covalent incorporation of methacrylated hyaluronic acid into a methacrylamide functionalized gelatin hydrogel, yielding the capacity to orthogonally manipulate hydrogel sti ness and HA content within the hydrogel. In addition to providing a platform to explore the effect of matrix-immobilized HA on GBM cell expansion, invasion, and response to therapeutic inhibitors (Chen et al., 2017; Pedron et al., 2017a), we recently demonstrated it was possible to incorporate vascular cells into the hydrogel, yielding complex vascular networks that remodeled over time (Ngo and Harley, 2017). While 3D organoid-type cultures have fostered the production of “human brain-like tissue” (Sasai, 2013; Lancaster and Knoblich, 2014), so far attention has focused primarily on forebrain cell groups (Birey et al., 2017; Quadrato et al., 2017) or traumatic brain injury (Tang-Schomer et al., 2014), yielding opportunities to explore.

This project set out to examine the translation of a HA-modified gelatin hydrogel for the *in vitro* culture of NGC neurons. To date, these neural cells have not been studied ex vivo, and given their relevance in linking brain development to behavior, a permissive culture system will allow neuroscientists systematically to sort out the separate contributions of extracellular matrix components and growth factors to their survival, growth and development. Here we report examination of the ability to grow NGC neurons *in vitro* in such hydrogels without the assistance of growth factors.

## MATERIALS AND METHODS

### Hydrogel Preparation and Characterization

Gelatin methacrylamide (GelMA) and HA methacrylate (HAMA) were synthesized as described previously (Pedron et al., 2015). The degree of functionalization (%DOF) was established by NMR as the ratio of methacrylamide groups (5.5 ppm) to aromatic groups (7.3 ppm). Freeze dried GelMA (3.5 wt%) was mixed with HAMA (0.5 wt%) into PBS containing 0.02% (w/v) lithium phenyl-2,4,6-trimethyl benzoyl phosphinate (LAP) as a photoinitiator until fully dissolved. Prepolymer solution was pipetted into Teflon molds 1.5 mm deep and 5 mm in diameter, and exposed to 10 mW/cm^2^ UV light (365 nm, LED AccuCure Spot) for 20 s.

Mechanical analyses were performed on fully swollen disks as described previously (Pedron et al., 2013). Specimens were tested in compression at room temperature via an Instron 5493 (100 N load cell) mechanical testing apparatus (20% strain/min). The compressive modulus was determined from the linear region corresponding to ~0–5% strain. Full laminin protein was replaced by CYIGSR sequence (generated at the Rockefeller University Proteomics Resource Center) previously described in the literature to support neuronal adhesion and growth (Mckinnon et al., 2013).

### Experimental Animals

C57BL/6/J mice (Jackson Laboratories) were maintained on a 12/12 light dark cycle with lights on at 7 a.m. and free access to water and food. Adult male and female animals were paired in the late afternoon to obtain timed pregnant dams and the morning when the formation of a vaginal plug was observed was counted as embryonic day 0. All experimental procedures were performed in accordance with the NIH guide for care and use of animals and approved by the Animal Care and Use Committee at The Rockefeller University. Pregnant females at 12 days post conception (E12) were sacrificed by cervical dislocation. After cleaning the abdomen with an excess of 70% ethanol, a V-shape cut was performed onto the abdomen through the skin and muscle layers to expose the peritoneum. The uterine horns were removed and transferred into a clean plastic dish containing ice-cold dissection buffer (DB) consisting of Hanks balanced salt solution, HBSS, Ca^2+^, Mg^2+^ free (Gibco), 20 mM D-Glucose (Sigma Aldrich) and 1% penicillin/streptomycin (Gibco). The average number of embryos per pregnant female was 6–8.

### Hindbrain Dissection and Primary E12 Hindbrain Cell Cultures

Under a dissecting stereomicroscope (Zeiss, model 10298), each embryo sac was separated from the intact uterine horns. The embryos were transferred to a clean plastic dish containing fresh ice-cold DB. The embryonic hindbrains were dissected according to Fantin et al. (2013) and the hindbrain dissociations were performed immediately after (see below). On average, the timing of dissection was 5 min per hindbrain.

### Hindbrain Dissociation

The collected hindbrains in DB were transferred to the tissue culture hood, and they were transferred to one of the wells of a 24-multiwell plate containing fresh DB. The dissection buffer was then replaced with 500 μl of the pre-warmed enzymatic cell dissociation reagent (StemPro^®^ Accutase, Thermo Fisher Scientific) and incubated at 37°C for 5 min with gentle shaking. The hindbrains were then mechanically dissociated pipetting up and down 12 times using a 1,000 μl tip and 12 more times using a 200 μl tip. Great care was taken in avoiding the formation of air bubbles during this step to maximize cell viability. The overall enzymatic and mechanical hindbrain dissociation step never exceeded 7 min. The enzymatic digestion was stopped by adding 3 ml of standard medium containing Dulbecco’s Modified Eagle’s Medium, DMEM (Gibco) supplemented with 10% fetal bovine serum (Gibco) and 1% penicillin/streptomycin (Gibco). The resulting cell suspension was filtered through a tube fitted with a cell strainer snap cap (35 μm nylon mesh, Falcon), transfer to a 15 ml Falcon tube and further diluted with standard media to a final volume of 10 ml. After gentle inversion, an aliquot of the suspension was mixed with an equal volume of trypan blue and the viable cell yield (cells/ml) was estimated using a Neubauer chamber. Immediately after, the volume of the suspension was adjusted with media to the desired cell concentration per gel and the samples were centrifuged at 200 g for 4 min at room temperature. After discarding the supernatant, the resulting cell pellet was immediately resuspended on the prepolymer hydrogel mixture (see section below).

### Encapsulation of Dissociated Embryonic Hindbrain Cells

We examined a library of hydrogel prepolymer solutions that varied by the overall total polymer concentration (5 or 4 wt%), the degree of methacrylamide functionalization of the gelatin macromer (DOF: 85, 60, or 55%), the fraction of HAMA(0.5 or 1 wt%) added to the GelMA solution, or the amount of LAP photoinitiator (0.05, 0.02 wt%) used to polymerize the hydrogel (Table 1). To promote the attachment of the embryonic hindbrain dissociated cells, a subset of hydrogels were functionalized with 800 μg/ml of a laminin peptide mimic (CYIGSR). The hydrogel components were dissolved in warm (45°C) neurobasal serum-free medium consisting of neurobasal medium, 2% B27 Supplement, 1% Glutamax, 1% penicillin/streptomycin. In one experiment, the components were dissolved in phosphate buffer saline as a control. All reagents were purchased from Gibco. The solution was kept at 37°C until the cell pellet was ready to be resuspended. After gently mixing, either 30 μl (10 million cells per ml) of the embryonic hindbrain cell suspension was pipetted onto Teflon molds anchored on top of a glass slide and secured with binder clips (Pedron et al., 2013, 2017a). Hydrogels were subsequently created: disks of 5 mm diameter; the height of the gels was 1.5 mm. The cell-laden prepolymer suspension was photopolymerized by exposure to 10 mW/cm^2^ UV light (365 nm) for 20 or 30 s (Table 1). Neurobasal medium was added to the gels immediately after polymerization to prevent dehydration. Each cell-seeded hydrogel was then transferred to a well of a 24-multiwell plate (Corning) containing neurobasal media and incubated at 37°C, 5% CO_2_ on top of the plate of an orbital shaker (lowest speed setting) inside a cell culture incubator. Neurobasal medium was exchanged every 3 days over the course of all experiments.

### Live/Dead Cell Viability Assay

The evaluation of cell viability was performed at 7 and 14 days *in vitro* (DIV) using the Live/Dead assay (Molecular Probes). After rinsing hydrogels with PBS (3× for 5 min each) they were incubated at room temperature with PBS containing 2 μM Calcein-AM (stains the cytoplasm of viable cells green) and 4 μM ethidium homodimer-1 (stains the nuclei of dead cells red). After 40 min, the cell-laden hydrogels were rinsed in PBS and immediately imaged using a confocal microscope (Zeiss LSM 880 inverted confocal laser scanning microscope). Because the hydrogel area exceeded an individual confocal image field we acquired the complete hydrogel image using the tile module included in the Zen confocal software (Zeiss). Starting at the surface and imaging down toward the center of the hydrogel, the first and last z positions for each z-stack/tile were defined for a total thickness of 100 or 200 μm. The acquired image tiles were then combined into one final output by a stitching process using a 10% tile overlap. For automated cell counts, and estimation of cell viability, the stitched output images were imported to the 3D rendering software Imaris (Bitplane). We used the spot detection module, and spots were defined using cell count parameters for size and fluorescence strength of voxels to represent each cell soma. Parameters were then subjected to multiple image tests between manually counted and automated cell counts in multiple regions of interest of the imported tiled images to ensure accuracy. Because the quantification of spots using 200 and 100 μm thick tiled z-stacks did not differ statistically within hydrogels, we did the analysis using the thinner tiled z-stacks images. After validation, parameters were saved and then applied through the entire 100 μm z-stacks and overall cell count data was obtained for each image. Cell viability was calculated as (number of green stained cells/number of total cells) × 100%.

### Immunocytochemistry

After 7 days *in vitro*, cell-laden hydrogels were rinsed 3× in PBS for 5 min per rinse and then fixed in 4% paraformaldehyde (Sigma Aldrich) for 5 min at room temperature. After rinsing with PBS, the gels were permeabilized with 0.5% Triton X-100 (Sigma Aldrich), blocked with 5% donkey serum (Invitrogen) and incubated overnight at 4**°**C with anti-TUJ1 mouse IgG primary antibody (1:1,000, Biolegend). Hydrogels were rinsed in PBS and then incubated for 2 h at room temperature with donkey anti-mouse IgG secondary antibody conjugated to Alexa Fluor 488 (Life Technologies, 1:1,000). Cellular nuclei were counter stained with 0.4 μl/ml DAPI in PBS for 5 min. The labeled hydrogels were examined and imaged using a Zeiss LSM 880 inverted confocal laser scanning microscope.

### Statistics

Statistical analysis was performed via one-way analysis of variance (ANOVA) tests after which a Tukey-HSD *post-hoc* test was used. Analysis of hydrogel mechanical properties used *n* = 5 constructs per group. Significance was set at *p* < 0.05. Error bars are reported as standard error of the mean unless otherwise noted.

## RESULTS

### Gelatin-Based Hydrogel Fabrication

Both the biomechanical and cell-adhesive properties of 3D hydrogel matrices are critical for the viability and behavior of encapsulated cultured cells. Initially, the conditions used for the encapsulation of E12.5 hindbrain cells comprised the use of 5 wt% total polymer concentration with an 85% degree of functionalization and 0.05 wt% photoinitiator. These conditions resulted in highly crosslinked polymer network that led to stiff hydrogels (8.7 ± 0.8 kPa) and proved to be highly detrimental to the survival and differentiation of the embryonic cells (data not shown). With the aim of designing a more compliant hydrogel, in subsequent experiments, we resorted to a hydrogel containing a lower total polymer concentration (4 wt%), lower degrees of functionalization of GelMA (60 or 55%), and photoinitiator concentration of 0.02 wt%. The more permissive conditions (1.1 ± 0.2 kPa) for cell viability resulted from platforms containing 55% DOF GelMA and 0.02 wt% concentration of photoinitiator. Interestingly, the addition of matrix immobilized HA to the GelMA hydrogel proved to be beneficial to sustain cell viability and differentiation, in some cases, of neuritic processes. The hydrogel composition employed in the present report consisted of GelMA/HAMA 3.5/0.5% and0.02% photoinitiator.

### Survival and Growth of Nucleus Gigantocellularis (NGC) Neurons

**FIGURE 1 |:**
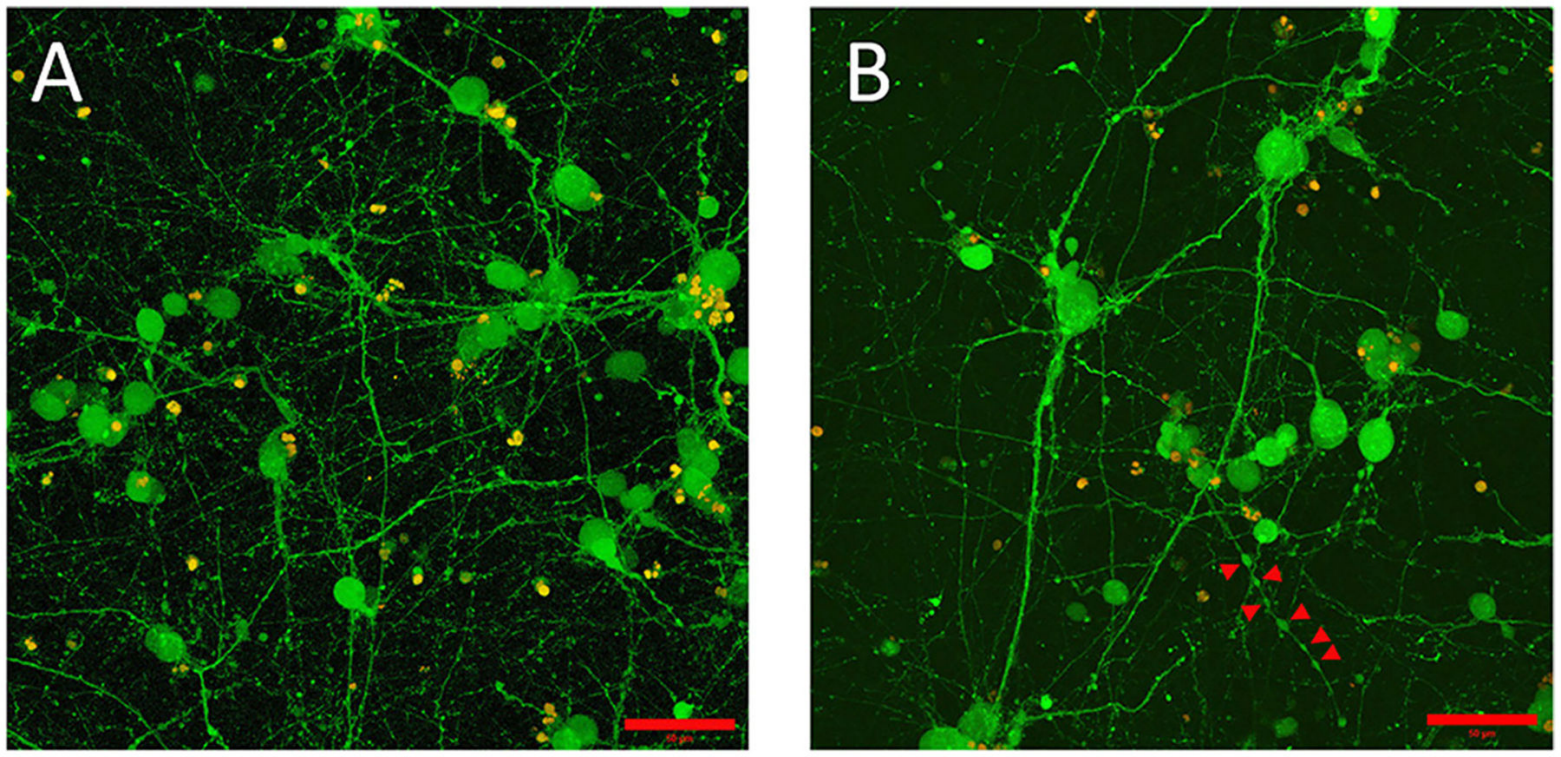
Embryonic 12.5 (E12.5) hindbrain cells encapsulated in 3D hydrogel scaffolds (GelMA/HAMA: 3.5/0.5 wt%, no laminin added) remained viable after 10 days *in vitro* (10DIV). **(A)** Maximum intensity projection depicts cell bodies projecting extensive processes adopting elaborated arborization patterns. Viable cells, shown in green, were labeled with calcein-AM (see Materials and Methods section for details). Scale bar: 50 μm. **(B)** Some encapsulated E12.5 hindbrain cells [same conditions as **(A)**] showed varicose neurites. Red arrows indicate the putative boutons along the neurite outgrowth. Scale bar: 50 μm.

**FIGURE 2 |:**
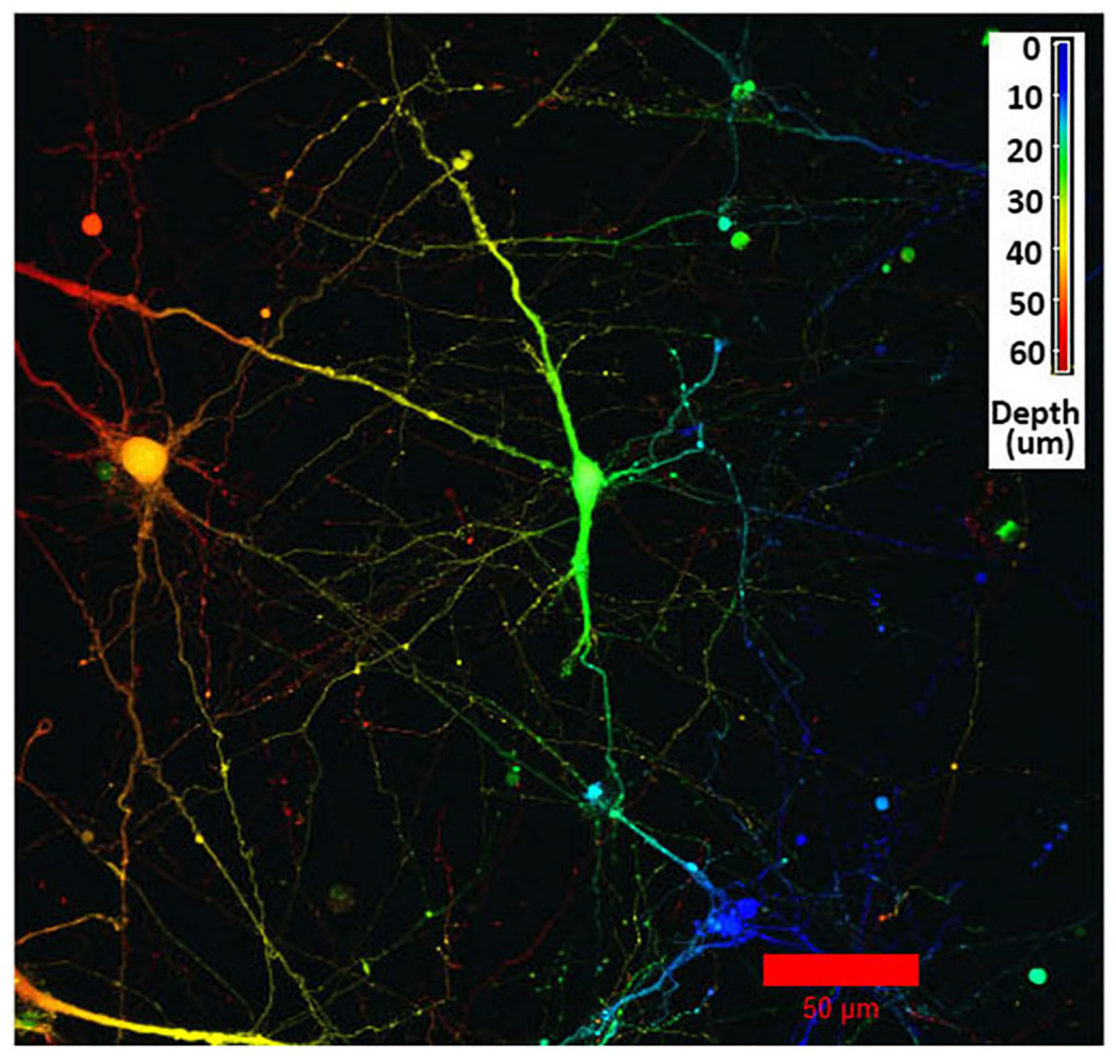
Confocal microscopy image using depth decoding of an E12.5 hindbrain cell hydrogel culture (GelMA/HAMA: 3.5/0.5 wt%, no laminin added) after 15DIV. Color decoding for the depth of the cells in the hydrogel along the z-axis is given. The difference in colors indicate the different planes along the z-axes. Scale bar: 50 μm.

The initial, pre-gel viability of these NGC neurons measured by dye exclusion was in the range of 60–85%. Cells encapsulated within the GelMA/HAMA (3.5/0.5 wt%) hydrogels and their associated neurite processes remained viable for up to 2 weeks *in vitro*. Cell viability at the termination of experiments was found to be between 30 and 60% as measured by the Live/Dead assay. Viable neurons usually sent out neurite processes, in some cases with the primary neurite branching and producing second-level or even third-level neurite segments (Figure 1A). This phenomenon was highly variable, both within and between gels. In some cases, neurites displayed ovoid varicosities along their length, with complex neuronal processes observed after 2 weeks *in vitro* (Figure 1B). Importantly, neurons were distributed three-dimensionally throughout the hydrogel, with Figure 2 depicting NGC cells extending processes (color coded by depth of penetration) into the hydrogel scaffolding.

**FIGURE 3 |:**
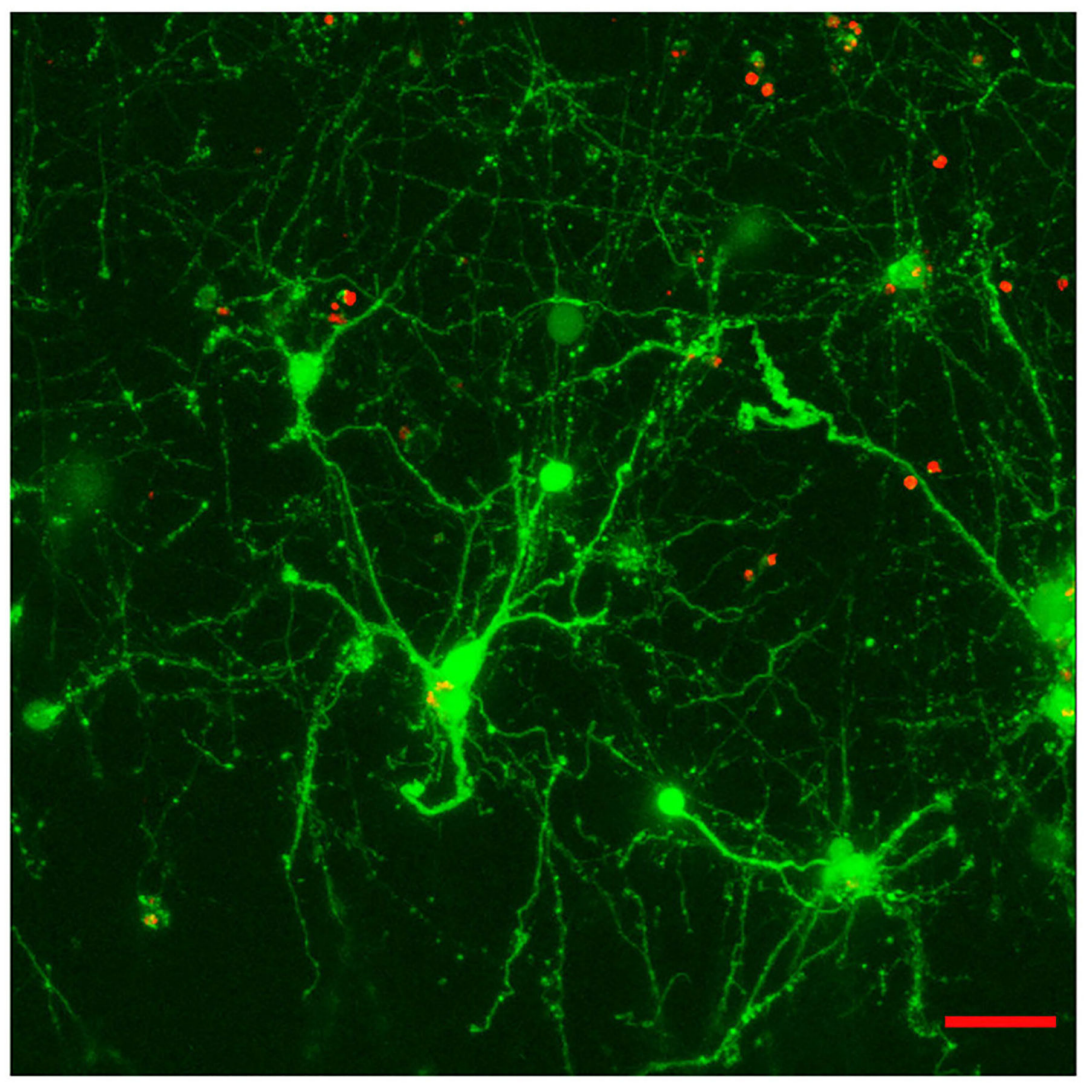
Effect of the incorporation of a cell-adhesive laminin peptide mimic into E12.5 hindbrain cell-laden hydrogels (GelMA/HAMA: 3.5/0.5 wt% plus 800 μg/ml CYIGSR) after 15DIV. Maximum projection of a 70 μm z-stack showing that encapsulated cells mature and give out neurites in the presence of laminin mimic. Scale bar: 50 μm.

**FIGURE 4 |:**
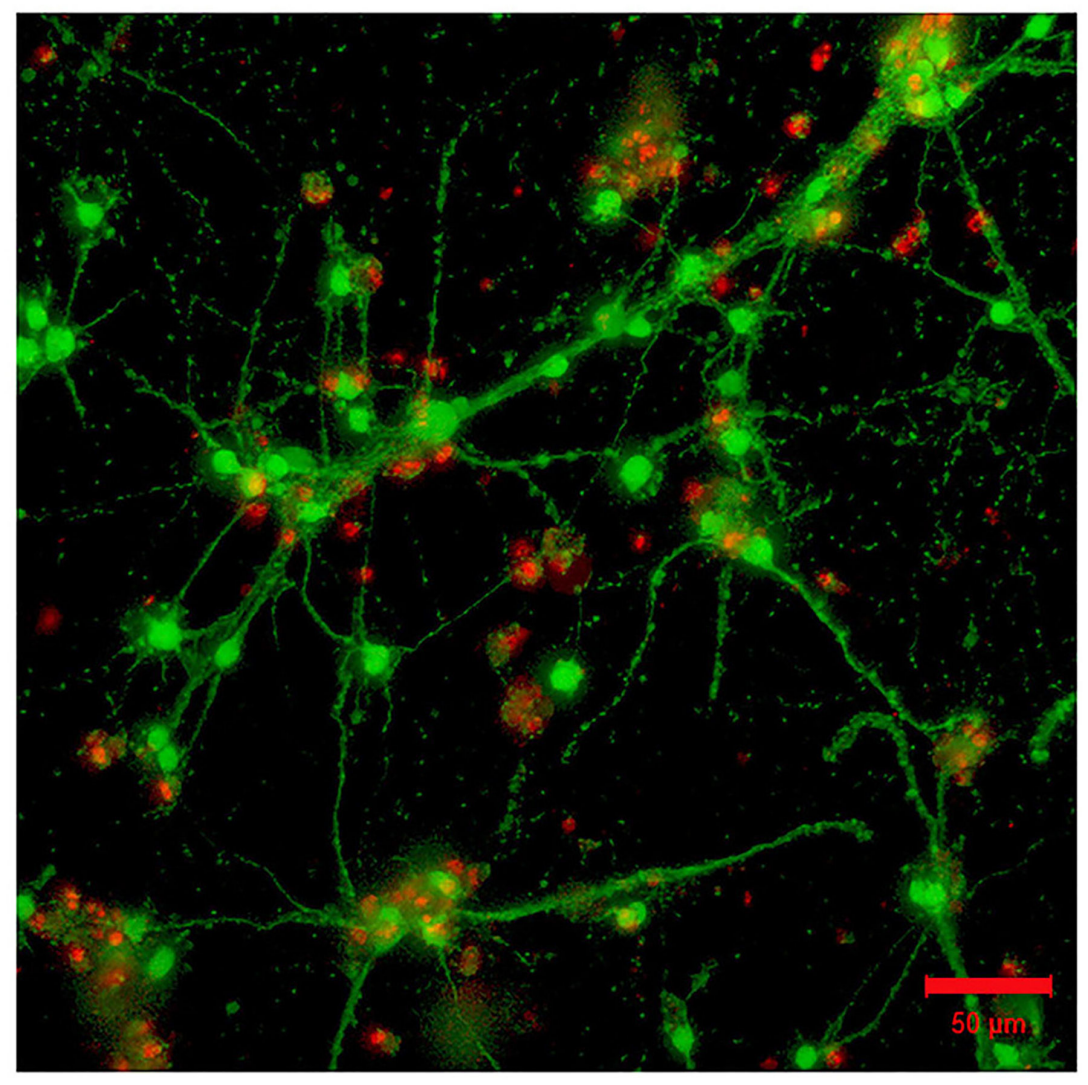
Absence of laminin in the 3D hydrogel (GelMA/HAMA: 3.5/0.5 wt%) is permissive for the outgrowth of neurites from encapsulated embryonic hindbrain cells after 15DIV. Scale bar: 50 μm.

Experiments did not include a systematic comparison of results with and without laminin. Instead, it must be said that, while it was possible to see robust neuronal network formation in hydrogels containing the laminin peptide (800 μg/ml) (Figure 3), morphologically similar results could be observed in the absence of laminin (Figure 4).

### Neuronal Differentiation of NGC Cells

**FIGURE 5 |:**
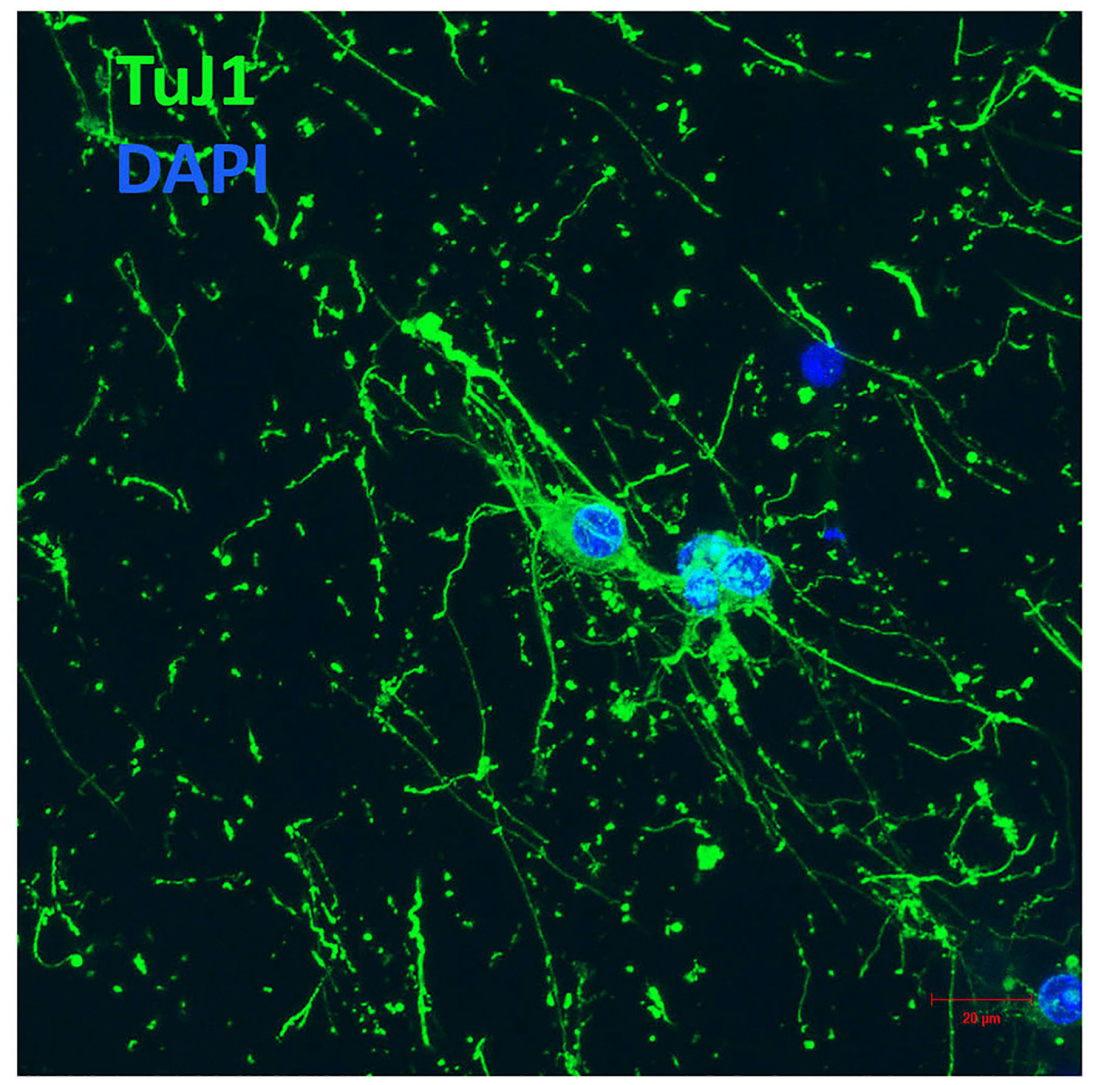
E12 hindbrain cells encapsulated in GelMA/HAMA: 3.5/0.5 wt% plus 800 μg/ml CYIGSR display a neuronal phenotype after 7DIV. Hydrogels were immunostained with Tuj-1, an antibody against neuron-specific class III-beta tubulin (see Materials and Methods section for details). Scale bar:20 μm.

We subsequently explored the initial stages of neuronal differentiation of NGC cells maintained within the HA-modified GelMA hydrogel. Excitingly, viable NGC cells adopted a neuronal morphology. Immunocytochemistry using an antibody against neuron-specific class III-beta-tubulin, a microtubule component used as a neuronal marker, confirmed that the cells encapsulated within the GelMA hydrogels displayed a neuronal phenotype (Figure 5). Further, after 7 days of culture in the hydrogels *in vitro*, cellular processes exhibited morphologies suggesting that they could form more complex neuronal networks.

## DISCUSSION

This work shows that it is possible to grow embryonic reticular formation neurons in 3D hydrogels in the absence of growth factors or nerve cell adhesion molecules. The neurons used, from the medial medullary reticular formation, which would include the large cells of nucleus gigantocellularis, are crucial for CNS arousal and the initiation of a wide range of behaviors. It remains to be seen whether optimum conditions for such neurons differ from those, for example, in the forebrain (Birey et al., 2017).

We selected gelatin-HA hydrogel as the base material for the fabrication of culture platforms for NGC neurons due to their success in hosting mammalian cells (Du et al., 2017), and our previous experience with cancerous glial cells (Chen et al., 2017; Pedron et al., 2017a,b). Biophysical properties of gelatin hydrogels can be easily manipulated by dialing concentration, degree of functionalization and photoreaction conditions (Pedron and Harley, 2013; Pedron et al., 2013, 2015, 2017a). Primary neural cells are very vulnerable to *in vitro* culture conditions and need very specific environmental conditions (e.g., mechanical stress, porosity, degradability, attachment sites) to keep them viable and functional. Very mild conditions of polymerization, low polymer concentration, and compliant resulting gels (Palazzolo et al., 2015) are known to be necessary for achieving a high initial cell viability, very important in the case of neurons that do not proliferate. In this case, the most permissive gels presented an elastic modulus around 1.1 kPa, and cells show long interconnected processes with the appearance of varicosities (Figure 1). In photo-initiated reactions, for instance, time, and intensity of exposure to UV light is a critical variable that needs to be considered during cell encapsulation that also engages material mechanical compliance. Ongoing work focuses on developing more compliant hydrogel constructs to ensure greater and more stable neuronal outgrowth. Indeed, matrix architecture is highly important in 3D microenvironments to allow for neurite extension at the same time as nutrients and oxygen flow. Current advancements in formation of endothelial networks within these constructs (Chen et al., 2017) could demonstrate as a prospective solution for the alteration of oxygen and biomolecule transport within the hydrogel network. Future directions aim to achieve a broader array of matrix architectures in order to optimize cell culture conditions: alternative crosslinking chemistries and simplification of sample fabrication by using spatially graded hydrogels (Pedron et al., 2017b) using microfluidics.

Despite our success in growing these neurons that are crucial for the initiation of behavior, several aspects of the work indicated that this methodology deserves further development. First, our 3D gels should in principle be amenable for electrophysiological recording. We note that it is harder to visualize the surfaces of target neurons through the gel than in a simple bath; and with the micropipette entering the gel from the right, advancing in the gel toward the target neuron pushes the gel (and the neuron) to the left even to the point where the neuron would be deformed. Second, despite exquisite attention to methodological detail, we noted variability in neuron viability and the consistency of neuron processes. In particular, we observed in some cases neurites presenting non uniform widths suggestive of varicosities or putative boutons. Although signs of axonal degeneration cannot be ruled out, future studies should address the latter possibility exploring the presence of vesicle associated proteins such as synapsin (Neto et al., 2014). In trying to explain the variability in neuron viability, one possibility lies with the method of neuronal concentration in the pellet of tissue immediately before adding the pre-polymer solution. Another possible cause of variability could stem from the crosslinking reaction and the hydrogel network formed thereof. Moreover, although adding laminin-derived adhesion peptide to the gel preparation did not systematically improve results, we had a small number of experiments in which the presence of the peptide mimic did improve viability and neuronal network formation, opening the door to exploring alternative methods to present adhesion moieties to NGC cells. One possible strategy to explore a wider range of combinations of adhesion molecules or a wider range of activity inducing factors would be the use of microfluidic patterning tools that we have previously described to create gelatin hydrogels containing embossed patterns of biomolecules (Pedron et al., 2013; Mahadik et al., 2015). Such tools can build on the observations here to test a wider range of concentrations, create patterns employing either or both soluble vs. hydrogelimmobilized signals, and would be ideally suited to examine a wider range of bioactive factors that might enhance neuron outgrowth that have been previously identified in the literature such as EGF and bFGF (Rao, 1999; Maric et al., 2003).

## CONCLUSION

For the first time, we report the successful *in vitro* culture and growth of primary NGC neurons, crucial for the initiation of behavior, within gelatin-based biomaterial platforms. HA-functionalized GelMA hydrogels, previously identified for the growth and expansion of glioblastoma cells, provides a 3D-hydrogel environment in which NGC viability, neuronal process extension, and the expression of markers of neuronal differentiation could be observed. Ongoing efforts are exploring modifications to the gelatin-based hydrogel biophysical properties to achieve a robust growth and stable neural differentiation for prolonged periods of time.

## Figures and Tables

**TABLE 1 |:** The composition (overall hydrogel wt %; GelMA/HAMA ratio; degree of methacrylamide functionalization of the gelatin macromer; inclusion of CYIGSR peptide), fabrication conditions (photoinitiator wt%; UV exposure time), and resulting elastic modulus (*n* = 4) of the family of hydrogel samples used in the encapsulation of NGC cells.

Total wt%	GelMA/HAMA %wt	DOF %	CYIGSR μg/ml	PI wt%	Time (s)	Modulus kPa
5	4.5/0.5	85	0	0.05	30	8.7 ± 0.8
4	3/1	60	0	0.02	20	5.0 ± 0.3
4	3/1	55	0	0.02	20	5.0 ± 0.3
4	3/1	55	800	0.02	20	
4	3.5/0.5	55	0	0.02	20	1.1 ± 0.2
4	3.5/0.5	55	800	0.02	20	
